# The Impact of Teacher Competence in Online Teaching on Perceived Online Learning Outcomes during the COVID-19 Outbreak: A Moderated-Mediation Model of Teacher Resilience and Age

**DOI:** 10.3390/ijerph19106282

**Published:** 2022-05-22

**Authors:** Yue Liu, Li Zhao, Yu-Sheng Su

**Affiliations:** 1 School of Education Science, Nanjing Normal University, Nanjing 210097, China; 200602149@njnu.edu.cn (Y.L.); li.zhao@njnu.edu.cn (L.Z.); 2 Department of Computer Science and Engineering, National Taiwan Ocean University, Keelung 202301, Taiwan

**Keywords:** online teaching, teacher competence, teacher resilience, teacher well-being, learning outcomes, COVID-19, teachers’ age

## Abstract

During the COVID-19 pandemic, teachers had to conduct online classes because of the breakdown of school learning. Teacher competence has a great impact on the students’ learning outcomes in online learning. Teacher resilience is also important to help teachers survive and achieve a high level of well-being in emergency situations. Previous studies have explored the protective and risk factors of teacher resilience, among which teacher competence in various aspects is included. In addition, teachers’ age differences in competence and resilience have been the focus of past studies. However, few studies have investigated the impact of teacher competence on students’ online learning outcomes, the mediating role of teacher resilience, and the moderating effect of age when teachers participate in emergent online teaching. To address the above gap, this study explored teachers’ perceptions of students’ online learning outcomes and how teacher competence in online teaching and resilience can predict these outcomes. The data of 159,203 participants were collected and subjected to correlation analyses and a moderated-mediation effect test. The results indicated that (1) teacher competence in online teaching was positively related to perceived online learning outcomes; (2) teacher resilience was positively related to the teachers’ perceived online learning outcomes; (3) teacher resilience played a partial mediating role between teacher competence in online teaching and perceived online learning outcomes; and (4) teachers’ age moderated the direct and indirect relation between teacher competence in online teaching and perceived online learning outcomes. The findings imply that teachers should strengthen their own teaching competence and their resilience before conducting online teaching. In addition, this study proposes intervention strategies to enhance teachers’ resilience and well-being through teacher competence cultivation and provides suggestions for different age levels of teachers to develop and train their online teaching competence and resilience in the future.

## 1. Introduction

During the COVID-19 pandemic, teachers have made use of digital technology for online teaching to bridge the time and space separation between teachers and students. Meanwhile, during the COVID-19 pandemic, teachers of all ages have also faced the core challenges of online teaching to varying degrees. The primary challenge is whether teachers are adequately equipped to use online teaching applications and ICT, that is, do they have online teaching competence [1]? Teachers’ professional competence greatly influences their teaching, as well as the academic achievement and future development of their students.

Recent empirical research suggests that teacher competence includes two areas of competence, which are as follows: one is cognitive competence and the other is motivational competence [2]. In terms of the cognitive area, it is emphasized that teachers should have certain types of knowledge, including pedagogical content knowledge (PCK), teachers’ content knowledge (TCK), and general pedagogical knowledge (GPK) [3]. With the application of digital technology in the education system, the usage of digital technology in the process of teaching has placed higher requirements on teachers’ competence. Accordingly, teachers’ knowledge and skills should be expanded and teachers should learn how to utilize technology effectively in their teaching. That is, teachers’ professional competence should be extended to their technological pedagogical knowledge (TPK) [1]. Teachers’ competence in the field of motivation mainly refers to their self-efficacy and teaching enthusiasm, which directly determine their teaching activities and professional engagement [4]. During the period of the COVID-19 outbreak, the implementation of online teaching not only tested teachers’ technological pedagogical knowledge (TPK), but also verified whether the current teachers’ self-efficacy and enthusiasm could adapt well to online teaching. Therefore, teacher competence in online teaching is particularly important in online teaching and learning, which is one of the important factors to help teachers master the core challenges during the period of the COVID-19 pandemic.

In addition, compared to traditional offline teaching, large-scale online teaching at home puts more pressure on teachers [5]. Due to the development of online teaching, in addition to teacher competence in online teaching, teachers’ stress, emotional experience, and emotional changes in online teaching due to difficulties in using digital technology, lack of online teaching experience, and poor conditions are the other core challenges that all teachers have faced during COVID-19 [6]. Teachers’ emotional control, pressure tolerance, and good emotional experience in the teaching process are the keys to overcoming the online teaching challenges during the COVID-19 pandemic [7]. Such a personality characteristic that moderates one’s stress and adapts positively in the context of significant adversity is called psychological resilience [8,9]. Teacher resilience is related to teachers’ well-being, which is considered to be the core of teachers’ professional life and an important factor in preventing job burnout [10,11]. Through research on middle school teachers, Brouskeli et al. [12] also found that teacher resilience is closely related to the five dimensions of teacher well-being. As Mansfield et al. [13] stated, teachers’ wellbeing is one of the important related outcomes of teachers’ resilience.

Being aware of the personal factors that support teachers’ resilience is a breakthrough to help teachers improve their well-being and prevent job burnout [14]. Gu and Day [15] confirmed that teacher resilience is influenced by personal factors, including self-briefing, emotional competence, self-efficacy, enthusiasm, and motivation. However, most studies on the personal influences on teacher resilience have been conducted in the context of formal offline teaching. Whether particular personal factors of teacher competence in online teaching will affect teachers’ resilience when they are faced with an unexpected large-scale online teaching situation has not been studied. Therefore, the focus of this study was to enhance teachers’ resilience and well-being through the intervention of cultivating teacher competence in online teaching. Furthermore, Day and Gu [16] stated that improving the quality of teachers’ teaching and students’ academic achievement requires a focus on building and maintaining teacher resilience. Thus, the relationship between teacher resilience and students’ learning outcomes in the process of online teaching were the main focuses of our study.

Prior studies have reported that online teaching significantly improved student learning outcomes compared to traditional teaching without the support of technology [17]. At the same time, it has been pointed out that using technology in teaching practice helps learners to learn easily, but whether effective learning outcomes can be achieved depends on teacher competence [18]. The differences in results suggest the need to study the mechanisms by which online teaching methods affect student learning outcomes, with particular attention to the impact of teacher competence on learners’ online learning outcomes. Based on online teaching, the roles of teacher competence in online teaching and teacher resilience in “special situations” are unclear. Specifically, as a socio-emotional regulation resource, resilience increases with age [19]. Past studies have tended to use age as a control variable affecting the results, but the possible differences in resilience and well-being among teachers of different age levels were ignored. Furthermore, few studies have explored whether the differences in teachers’ age might affect the mediating role of teacher resilience in the relationship between teacher competence in online teaching and perceived online learning outcomes.

To address the above gaps, this study evaluated students’ learning outcomes from teachers’ perspectives to explore whether teacher competence in online teaching can support students to achieve the desired learning outcomes in the context of a fully online environment, as well as whether teachers’ resilience in the face of emergent home-based online teaching will affect the above-mentioned pathway. Meanwhile, whether the impact of teacher competence in online teaching on perceived online learning outcomes via teacher resilience may be buffered by teachers’ age is another focus of this present study. The findings can provide a theoretical basis for better developing teachers’ multifaceted competencies in teacher education and building sound teacher resilience when teachers face unexpected situations. At the same time, corresponding interventions and policies are provided to help teachers of different age groups adapt to the wide application of digital technology in education, improve their resilience, and enhance their well-being.

## 2. Theoretical Background and Hypotheses

### 2.1. Teacher Competence in Online Teaching (TCOT) and Perceived Online Learning Outcomes (POLO)

Based on the existing research about online teaching, online teaching is summarized as differing from conventional teaching in terms of the following four characteristics: (1) long-distance communication between teachers and students is carried out through a digital platform; (2) relevant learning materials are accessed through technology; (3) teachers and students use technology to interact with each other; and (4) teachers use digital communication channels to assist students in learning [20]. From the concept discrimination of online teaching, for teachers, it is not easy to carry out online teaching well if they are not equipped with competent pedagogical knowledge and technological competence. Specifically, the present study paid attention to TCOT during the COVID-19 outbreak.

Generally speaking, competence refers to a set of knowledge, skills, and experience that are used to meet one’s needs in the future [21]. Expanding to the concept of teacher competence, it refers to some specific qualities that teachers possess to satisfy their high professional demands, and is composed of two aspects of competence, which are as follows: the cognitive aspect and the motivational aspect [22,23]. In terms of teachers’ cognitive aspects of competence, most studies have focused on teachers’ knowledge (e.g., pedagogical content knowledge (PCK) and technological pedagogical knowledge (TPK)) and their beliefs [24,25]; the structure of teachers’ competency in the motivational aspect is composed of self-efficacy [26] and teacher enthusiasm [27].

From the cognitive aspects of teacher competence, it has an important influence on teachers’ professional development [28] and is even more relevant to students’ academic outcomes [29,30,31]. Huma et al. [32] stated that teachers’ content knowledge facilitates learners’ understanding of relevant concepts encountered in the learning process, and student learning outcomes are maintained through attention to and training of teachers’ content knowledge. A strong correlation between teachers’ PCK and students’ academic achievement has been established [29,31]. Furthermore, this study was based on the belief that one of the biggest differences between online learning and offline teaching is the usage of ICT technology. In the period of the COVID-19 pandemic, both teachers and students were forced to turn to online learning and teaching, which places higher requirements on teachers’ competence, especially their technological pedagogical content competence (TPCK). Prior studies have focused on how teachers use technology tools to carry out online teaching, and how ICT enhances students’ online learning [33,34]. Therefore, teachers’ TPCK also has a great influence on improving students’ learning effectiveness. As for teachers’ beliefs, previous studies have shown that they influence teachers’ integration of technology into their teaching [35], thus affecting students’ learning outcomes in many aspects [26].

As for the motivational aspect, most studies evaluate the motivation aspect of teacher competence by measuring their self-efficacy and enthusiasm. According to Bandura’s [36] social learning theory and research on teachers’ self-efficacy, teachers’ confidence in their ability to successfully complete teaching tasks in a specific situation is defined as teacher self-efficacy. Some studies have confirmed that teachers’ self-efficacy, which is a unique characteristic of teachers, is the decisive ability to help teachers adapt to online teaching [1,37]. In particular, studies have pointed out that in online teaching, the higher the level of self-efficacy that teachers have, the higher their teaching satisfaction will be, and students can show positive learning results [38]. Teachers’ teaching enthusiasm is also an important dynamic factor affecting teachers’ responses to daily classroom teaching challenges, and has a significant impact on students’ learning interest [39], which is an important evaluation index of students’ learning outcomes.

For online teaching supported by ICT technology, this study should not only pay attention to the impact of technology itself on teaching, but also to whether teachers are competent in technology-assisted teaching. It has been pointed out that only when online learning can be carried out effectively can learners have high levels of satisfaction, and student satisfaction is one of the important evaluation indexes of students’ online learning outcomes [40,41]. Prior studies have proved that teacher competence may indirectly affect students’ learning outcomes through teaching quality [42,43]. Through a longitudinal study, Shin and Shim [44] indicated that teacher competence not only affects student engagement, but also has a significant correlation with students’ learning achievement. Some studies have also pointed out that teachers need to effectively manage the teaching process to help students learn and use the knowledge they have learned, so teachers need to develop their competence according to the educational activities. Based on such reasoning, teacher competence might have a significant impact on the improvement of students’ learning achievement [45]. Based on the above arguments, the following hypothesis was proposed:

**Hypotheses** **1** **(H1).** *Teaching Competence in Online Teaching (TCOT) is Positively Related to Perceived Online Learning Outcomes (POLO).*

### 2.2. The Mediating Effects of Teacher Resilience (TR)

As one of the important psychological structures affecting teachers’ well-being [46], teacher resilience comprises many factors that can help individuals survive in adversity, such as self-esteem, self-efficacy, and motivation. The teachers who possess resilience are able to regulate their negative emotions, generate positive emotional experiences, such as feelings of pride and achievement, and effectively deal with pressure to grow in difficult situations [7,47,48]. Through the interpretation of the concept of resilience and teacher resilience by different scholars, all the definitions emphasize that teacher resilience reflects the adaptation to the environment and growth in adversity [49,50,51]. For teachers, launching large-scale online teaching is undoubtedly an adversity full of challenges.

During the COVID-19 pandemic, teachers have had to provide online teaching to students. On the one hand, online teaching consumes a great deal of time, adding pressure to teachers’ daily life. On the other hand, teachers are required to master technical skills, which gives them some technical pressure. The existing research results regarding teachers with online teaching experience show that teachers experience a certain level of stress when participating in online teaching [6,52,53]. During the period of COVID-19, developing online teaching has been mandatory to some extent, which is different from the spontaneous behavior of teachers, resulting in a stronger sense of stress. A review on teacher professional development confirmed that dilemmas and conflicts will arise when teachers need to deal with urgent issues of digitization [54], intercollegiate collaboration, and research-based teaching methods. A prior study on teacher resilience suggested that it can be stimulated and nurtured during periods of stress [55], and resilience strategies can be used to support the development of teachers’ well-being [56]. Therefore, the development of teachers’ resilience in the face of the pressure brought by the abrupt change of teaching model and the application of technology in teaching during the epidemic deserves our attention.

In the face of the existing challenges, it is necessary to explore what factors contribute to the development of teachers’ resilience, thus, helping teachers survive and grow in emergency situations. A large number of empirical studies have adopted qualitative research methods to confirm that individual and contextual factors have impacts on teacher resilience [57]. These factors include school leaders, colleagues, relationships with students, family support, and so on [58]. Compared with complex contextual factors, the influence of teachers’ personal factors on their resilience is more significant. Through the research on teachers’ resilience, a consensus has been reached; at the individual level, teachers’ motivation and other abilities will help teachers to show higher resilience [49,59]. Some studies have pointed out that teachers’ self-efficacy should be developed in order to promote their resilience. Facing setbacks and adversity, teachers with high levels of self-efficacy will show higher resilience, allowing them to be adaptive to changes in the environment [15]. In the past, qualitative research methods were used to explore the personal protective factors of teachers’ resilience, that is, what personal protective factors would help teachers maintain resilience when facing adversity. These personal factors include emotional intelligence, self-efficacy, intrinsic motivation, enthusiasm, teaching skills, and so on [60]. Similarly, studies have pointed out that teacher resilience can be influenced by personal protective factors at the professional level, such as teachers’ pedagogical competence [61]. The factors such as lack of competence and self-efficacy make teachers’ resilience and well-being deteriorate in distance teaching during the COVID-19 pandemic [62]. The abovementioned personal protective factors of teacher resilience can be summarized as various attributes of teacher competence. Along this line of reasoning, teachers with higher teacher competence in online teaching can produce higher levels of teacher resilience to better overcome the adversity of online teaching. Thus, H2 was proposed, which is as follows:

**Hypotheses** **2** **(H2).** *Teaching Competence in Online Teaching (TCOT) is Positively Related to Teacher Resilience (TR).*

Prior studies have shown that teachers with high levels of resilience can bring students enjoyment in the classroom [7]. Positive psychological resilience or lack of resilience not only affects teachers’ own professional happiness but also students’ learning outcomes [63]. Teachers serve as role models for students. If teachers do not demonstrate the quality of resilience, students will not be able to be resilient [64]. Resilient teachers can create a positive classroom atmosphere in which learners can enjoy learning and can make progress academically [65]. Thus, this study proposed H3, which is as follows:

**Hypotheses** **3** **(H3).** *Teacher Resilience (TR) is Positively Related to Perceived Online Learning Outcomes (POLO).*

Teacher resilience refers to the ability to recover quickly when faced with adversity. Resilient teachers are described as people who have competence in difficult situations, the skill of behavior management, the ability of empathizing with difficult students and suppressing negative emotions, and who experience pride and achievement in order to better complete teaching tasks and help students achieve their learning goals [48]. Teacher competence (i.e., self-efficacy, enthusiasm, etc.) enables teachers to recover quickly from adversity, which in turn promotes students’ learning achievements [50]. Therefore, teachers’ competence can directly affect students’ learning results, and can also indirectly affect children’s learning through teachers’ resilience. It is closely related to teachers’ self-efficacy, motivation, and other teacher competences, which are conducive to promoting students’ learning achievements. During the COVID-19 pandemic, the sudden transformation from offline teaching to online teaching on a large scale required teachers to be resilient to help students achieve the learning outcomes of online learning. Highly resilient teachers should have the competence to respond to teaching emergencies. Thus, the following hypothesis was proposed:

**Hypotheses** **4** **(H4).** *Teacher Resilience (TR) Mediates the Relation Between Teaching Competence in Online Teaching (TCOT) and Perceived Online Learning Outcomes (POLO).*

### 2.3. The Moderating Effect of Teachers’ Age (TA)

Although teacher competence in online teaching may improve online learning outcomes through teacher resilience, it seems that not all teachers would experience poor resilience leading to the poor learning performance of their students when they lack competence in online teaching. This situation may be caused by teachers’ age. According to the socio-emotional selectivity theory, as individuals grow older, their physical health and cognitive ability will decline, while their emotions and well-being remain at a high level [66]. Studies about the effect of teachers’ age on teacher competence are inconclusive. It has been pointed out that teacher competence differs by age. For example, Zyad [67] found that younger teachers were more likely to use ICT in their teaching than older teachers. Likewise, Krumsvik et al. [68] concluded that the older teachers were, the less digital competence they would have. However, contrasting results were obtained by Semerci and Aydin [69], whose study confirmed that there were no significant age differences in teacher competence in ICT usage. The change in teachers’ resilience as they age has also been explored in a number of studies. For instance, Reed [70] pointed out that resilience increased reliably with individuals’ age. Scheibe et al. [71] reported that older university workers showed greater resilience than younger workers during the COVID-19 pandemic. Therefore, the effect of teachers’ age on the relationship between teacher competence in teaching and their resilience or well-being should be explored.

Furthermore, teachers’ age may play a moderating role in the direct relation between teacher competence in online teaching and perceived online learning outcomes, as well as the indirect effect of resilience. Studies have indirectly shown the tendency that teachers’ teaching effectiveness being easily influenced by their competence is more significant among older teachers. For example, Tiraieyari and Uli [72] revealed that the relationship between social competencies and work performance was stronger among older than among younger participants. Therefore, it can be inferred that TA may strengthen the association between TCOT and POLO.

There is no direct evidence to prove whether there are differences in the impact of teachers’ resilience on students’ learning outcomes among different teacher age groups. However, the weakening effect of stressors on workers’ job performance with increasing age was confirmed in Shirom et al.’s research [73]. Resilience is used to counteract the negative effects of stress on people [74]. Stress had a lower impact on the job performance of older workers, suggesting that resilience provided better protection. Therefore, we hypothesized by analogy that age weakens the relationship between stressors and job performance, but significantly strengthens the association of the positive effect between TR and POLO.

As for the moderating role of age in the associations between teacher competence in online teaching and their resilience, indirect evidence has been provided in prior studies. Studies have shown that older teachers are not as competent as younger teachers in the online teaching process [68], but their resilience level is higher [71]. However, studies have also indicated that the higher the level of competence, the higher the level of resilience. Considering such contradictory arguments, it may be that TA weakens the relationship between TCOT and TR. This inference is consistent with Zacher and Schmitt’s literature review [75], which suggested that the relation between work characteristics and occupational well-being was strong among older workers.

Furthermore, the existing studies found that older teachers who performed poorly in teacher competence in online teaching [76] had accumulated more resources for resilience in the past [77], which helps their students acquire more positive learning outcomes [78]. In addition, combining the person-environment fitting theory and socioemotional selectivity theory, it can be inferred that personal factors, including characteristics and competence, are related to age, and such changes will bring changes in well-being to people [66,79]. Therefore, we inferred that the mediating effect of resilience in the relationship between TCOT and POLO would be stronger in older teachers.

Thus, based on the above discussion, we proposed H5, which is as follows:

**Hypotheses** **5** **(H5).** *Teachers’ Age (TA) Moderates the Direct and Indirect Relation Between Teaching Competence in Online Teaching (TCOT) and Perceived Online Learning Outcomes (POLO).*

### 2.4. Hypothesized Conceptual Model

**Figure 1:**
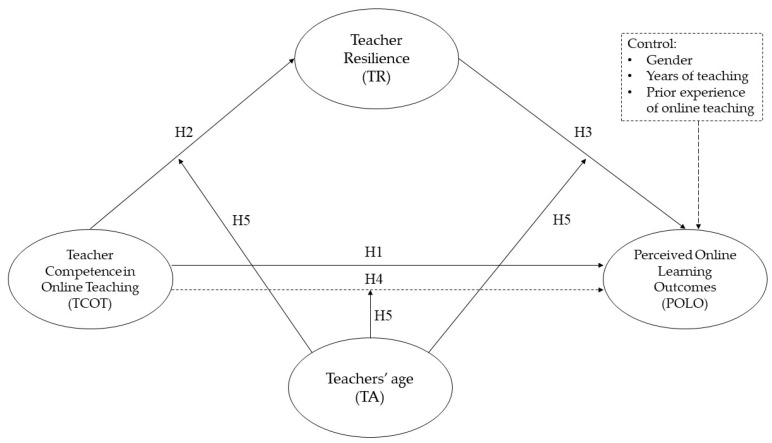
The moderated-mediation model. (Source: drawn up by the authors).

The person-environment fit theory proposes that stress derives from the mismatch between job requirements and personal competence [80,81]. In the period of the COVID-19 outbreak, the sudden transition to large-scale online teaching required teachers to quickly adapt to online teaching, and accordingly required them to develop teacher competence in online teaching. Faced with this mismatch, teachers experienced a certain amount of stress, and coping with this stress was an important reflection of teacher resilience. Based on the aforementioned theoretical streams of research and the person-environment fit theory, this study explored the links among teacher competence in online teaching, teacher resilience, and student online learning outcomes, as well as the moderating role of teachers’ age. Accordingly, the research model of this study is illustrated in Figure 1. It has been found that men and women show significant differences in their knowledge, abilities, and attitudes toward technology use [32,82]. The present study was concerned with teacher competence in online teaching contexts, which involves the usage of digital technology. It was, therefore, necessary to control the effect of their gender differences on the results. Moreover, it has been indicated that older teachers have lower levels of competence than younger teachers [83,84], so prior teaching experience is another important factor that may affect teacher competence [85].

Accordingly, based on previous research, teachers’ gender and years of teaching were included as control variables when testing the moderated-mediation effect.

## 3. Methodology

### 3.1. Participants and Procedure

The target population of the present study was elementary and secondary school teachers who used digital teaching tools to teach students remotely during the COVID-19 closure in China. The teachers covered a wide range of subjects, grade levels, areas, ages, and genders. Some of them had prior experience of online teaching, while for others, it was their first time using online teaching tools to carry out their teaching activities.

Online questionnaires were distributed via Questionnaire Star (www.wjx.cn) (accessed on 28 July 2021), a professional online survey tool widely used in China. The link to the online questionnaire was sent to local elementary and secondary schools, which in turn sent the link to the teachers, ensuring that the questionnaires were filled out without duplication or omission. The anonymity and voluntariness to participate in this survey, as well as the confidentiality of collected data and information, were stated to the participants in the preamble of the questionnaire before they filled out the questionnaire. Eventually, 180,965 teachers, who were told that the data would not be used for other purposes but limited to use in this study, took part in the study by completing the online questionnaire.

### 3.2. Instrument

To measure the variables, the teacher competence scale, teacher resilience scale, and online learning outcomes scale were used in our questionnaires. All were 5-point Likert scales. Demographic information about the respondents was collected in the first part of the questionnaire, including gender, age, discipline, years of teaching, and so on.

#### 3.2.1. Teacher Competence in the Online Teaching Scale

To measure teacher competence in online teaching, a questionnaire with the following four dimensions was applied: teachers’ technological pedagogical knowledge (TPCK), belief, self-efficacy, and enthusiasm. The items for this measurement were mainly adapted from Lange’s PCK Scale [86], Warwas, Hertel, and Labuhn’s Constructivist Beliefs Scale [87], Schmitz and Schwarzer’s [88] Self-Efficacy Scale, and Kunter’s Teaching Enthusiasm Scale [89]. The scale of teacher competence consisted of 12 items rated on 5-point options (from “(1) never” to “(5) always”), where the higher the score, the higher their level of teacher competence in online teaching. For example, “I can select appropriate teaching methods for the content of the online teaching.”

#### 3.2.2. Teacher Resilience Scale

As one of the important psychological structures of teacher well-being, teacher resilience is an important evaluation index to predict teacher well-being [48]. Therefore, teachers with high resilience showed a high level of well-being. The items for teacher resilience were mainly adapted from Mansfield and Wosnitza’s [90] TRSR Scale, which assesses the protective and risk factors related to teacher resilience. Moreover, this study focused on teachers’ resilience in online teaching, so the present study paid more attention to teachers’ emotional control and tolerance of online teaching stress. Therefore, the teacher resilience scale was composed of 5 items in the form of 5-point options (from “(1) never” to “(5) always”), with lower scores indicating a high level of teacher resilience and well-being. For example, “When parents, colleagues and principals see my online classes, I feel the pressure of being ‘monitored’”.

#### 3.2.3. Perceived Online Learning Outcomes Scale

The items for teacher perceived online learning outcomes of students in this study were mainly adapted from the learning outcomes tool established by Bae [91], which assesses the learning outcomes of students in distance e-learning. To make the items more in line with the online teaching background, the items were properly modified. Meanwhile, as the participants of this study were teachers and this study measured the teachers’ perceptions of students’ learning outcomes, the items were adjusted accordingly. Finally, the perceived online learning outcomes scales were composed of 6 items in the form of 5-point options, ranging from “strongly disagree” to “strongly agree”, for example, “I think my students can adapt to online learning very quickly.”

### 3.3. Reliability and Validity Test of Scales

The internal consistency was evaluated and indicated by Cronbach’s α. The Cronbach’s α reliability coefficient of teacher competence in online teaching scales was 0.942, that of the teacher resilience scale was calculated as 0.825, and that of the perceived online learning scale was 0.887, indicating that the collected data of this study were valid and reliable [92]. The construct validity of this questionnaire was ensured by exploratory factor analysis (EFA). The value of KMO was 0.932 (*p* < 0.001), indicating the feasibility of the factor analysis. Then, the varimax-rotation method and principal component analysis (PCA) were used. The results indicated that all the items loaded significantly at 0.50 among all the factors. The amount of data collected in this study was substantial so that it could fit the conceptual model. Although CFA was not performed, subsequent data analysis and mediation effect analysis can still be carried out at some time in the future (e.g., [93]).

### 3.4. Common Method Biases

Common method bias of this study was tested by Harman’s single-factor test, and an exploratory factor analysis was run to ensure the reliability and validity of all items. The results indicated that 4 factors’ eigenvalues were all greater than 1, and the variation explained by the first factor accounted for 21.837%, which is below the critical value of 40% [94]. By extrapolation, there was no problem of common method bias in this study.

### 3.5. Data Analysis

Data analysis was performed using IBM SPSS Statistics, version 25.0 (Armonk, NY, USA). To verify the hypotheses, descriptive statistics including mean value and standard deviation, correlation analysis, and ANOVA analysis were conducted. Then, to further confirm the relation among teacher competence in online teaching, teacher resilience, and students’ online learning outcomes, the mediation model provided by the SPSS macro PROCESS version 2.15 (model 4) was adopted, as suggested by Hayes [95]. Finally, model 59 in PROCESS was applied to analyze the moderating role of teachers’ age. We performed a 10,000 Bootstrap test to examine the mediator effect. If the confidence interval (CI) does not include zero, the moderated-mediation effects can be considered as significant.

## 4. Results

### 4.1. Descriptive Analysis

After data screening and omitting outliers, incomplete responses were deleted and a total of 159,203 responses were obtained with effective recovery of 87.97%. The respondents consisted of 111,748 females (70.2%) and 47,455 males (29.8%), with 3729 (7.9%) having 1–3 years of teaching experience, 2824 (5.9%) with 4–6 years, 2699 (5.7%) with 7–10 years, and 38,203 (80.5%) with over 10 years. While 133,588 of the respondents were teaching the major subjects of Chinese (*n* = 51,080, 32.1%), mathematics (*n* = 39,726, 25.0%), English (*n* = 25,173, 15.8%), physics (*n* = 5338, 3.4%), chemistry (*n* = 3638, 2.3%), geography (*n* = 2347, 1.5%), biology (*n* = 2620,1.6%), and history (*n* = 3666, 2.3%), others were teaching other subjects including PE, music, arts, and so on (*n* = 25,615, 16.0%). The online teaching experience of teachers was also checked and it was found that 4% (*n* = 6355) of respondents indicated that they did not have any experience of online teaching and knew nothing about online education, while 20.0% (*n* = 31,860) did not have online teaching experience but they had certain knowledge of online education, 35.1% (*n* = 55,839) had several instances of online teaching experience and they knew about online education, and 40.9% (*n* = 65,149) had accumulated some online teaching experience. As for teachers’ age, 6.7% (*n* = 10,708) of teachers were under 25 years old, 32.7% (*n* = 52,053) were from 26 to 35 years old, 37.5% (*n* = 59,651) were from 36 to 45 years old, and 23.1% (*n* = 36,791) were over 45 years old.

### 4.2. Correlational Analysis

ANOVA was used to examine the differences in gender (male and female), years of teaching (1–3, 4–6, 7–10, ≥11), prior experience of online teaching (never, sometimes, often, usually) and teachers’ age (≤25 years old; 26–35 years old; 36–45 years old; ≥46 years old) between the TCOT variable and the TR variable. The results indicated significant differences between male and female teachers in TCOT (*p* < 0.001) and TR (*p* < 0.001). In particular, female teachers showed higher teacher competence in online teaching, but male teachers showed higher teacher resilience in comparison to female teachers. In addition, teachers’ years of teaching also had an effect on TCOT (*F* (3, 159,199) = 5.648, *p* < 0.05) and TR (F (3, 159,199) = 211.288, *p* < 0.001). The post hoc tests indicated that teachers with 1–3 years of teaching experience showed poor teacher resilience compared with other teachers, while there were significant differences between teachers with over 11 years of teaching experience and other teachers, with the more experienced teachers showing poor teacher competence in online teaching. Finally, there is an age difference in TCOT (*p* < 0.001) and TR (*p* < 0.001). The post hoc tests indicated that young teachers (≤25 years old) showed a higher level of teacher competence in online teaching compared with other teachers, while the teachers aged 36–45 experienced higher levels of teacher resilience than other teachers.

For the results of the correlation analysis of the variables, see Table 1. Teacher competence in online teaching was positively correlated with teacher resilience (*r* = 0.323, *p* < 0.01) and teacher perceived online learning outcomes (*r* = 0.407, *p* < 0.01). Teacher resilience was positively related to teacher perceived online learning outcomes (*r* = 0.296, *p* < 0.01). Teachers’ age was negatively related to teacher competence in online teaching (*r* = −0.094, *p* < 0.01) and teacher perceived online learning outcomes (*r* = −0.059, *p* < 0.01). Noteworthily, according to the *r*-value, the correlations between teachers’ age and teacher competence in online teaching, as well as teachers’ age and teacher perceived online learning outcomes were small in magnitude. Teachers’ age had no significant relation with teacher resilience in online teaching (*r* = −0.004, *p* > 0.05).

### 4.3. The Mediating Role of Teacher Resilience

Model 4 from the SPSS macro PROCESS version 2.15 was used to verify the proposed mediation model, with teacher resilience as the mediator. After controlling for teachers’ gender, teaching age, and their prior experience of online teaching, teacher competence in online teaching was positively associated with perceived online learning outcomes without the mediator (*β* = 0.370, *p* < 0.001). As observed in Table 2, when teacher resilience was included, teacher competence in online teaching was positively associated with teacher resilience (*β* = 0.420, *p* < 0.001), and was positively associated with perceived online learning outcomes (*β* = 0.373, *p* < 0.001); thus, H1 and H2 were supported. Teacher resilience was positively associated with perceived online learning outcomes (*β* = 0.161, *p* < 0.001); thus, H3 was supported. When the mediator was included, it was considered significant that through teacher resilience, teacher competence was an indirect predictor of perceived online learning outcomes. Teacher resilience partially mediated the relation between teacher competence in online teaching and perceived online learning outcomes; thus, H4 was supported. The results above indicate that there is significant mediation between teacher competence in online teaching, teacher resilience, and perceived online learning outcomes. As H4 showed, teachers with higher teacher competence in online teaching reported a higher level of resilience, which in turn led to an increase in students’ learning outcomes. The coefficient and 95% CI of each indirect effect, total effect, and direct effect in the mediator model are shown in Table 3. The direct effect (0.374) accounted for 84.81% of the total effect (0.441); meanwhile, the indirect effect (0.067) accounted for 15.19%.

### 4.4. The Moderating Role of Teachers’ Age

**Figure 2:**
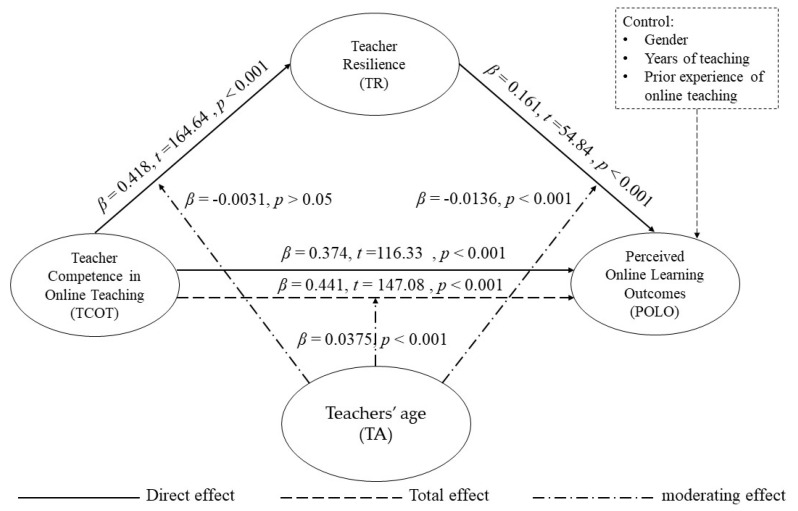
Moderated-mediation role of teachers’ age. (Source: drawn up by the authors).

The moderated mediation analysis was tested using Model 59 from the SPSS macro PROCESS version 2.15 (see Figure 2) [95]. Teachers’ age was taken as the moderating predictor, and the result is shown in Table 4. The effect of TCOT on POLO was moderated by teachers’ age (β = 0.0375; *p* < 0.001), and the effect of TR on POLO was also moderated by teachers’ age (β = −0.0136; *p* < 0.001), while the association between TCOT and TR was not significantly moderated by TA (β = −0.0031; *p* > 0.05). That is, TA can significantly moderate the relationship between TCOT, TR, and POLO. Thus, H5 was partly supported.

**Figure 3:**
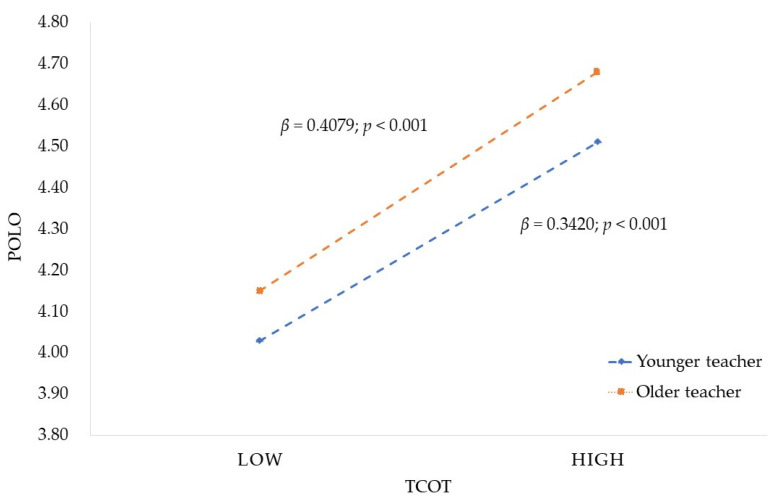
Moderating role of TA in the relationship between TCOT and POLO. (Source: drawn up by the authors).

**Figure 4:**
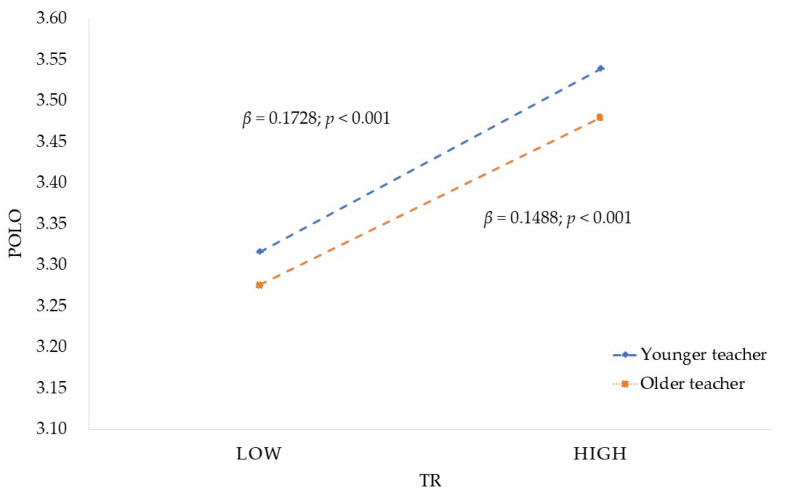
Moderating role of TA in the relationship between TR and POLO. (Source: drawn up by the authors).

The simple slopes analysis [96] result is shown in Figure 3 and Figure 4. As the age of teachers increases, the relation between TCOT and POLO strengthens, while the relationship between TR and POLO weakens. That is, for older teachers (M + 1SD, about age > 45) [97], the impact of TCOT on POLO was stronger (β = 0.4079, *p* < 0.01), while for younger teachers (M − 1SD, about age < 35) [97], the impact of TR on POLO was stronger (β = 0.1728, *p* < 0.01). As shown in Table 5, the indirect effect of TCOT on POLO through TR was also conditioned by TA. The conditional indirect effect on different age levels of teachers was always significant. However, it was weakest for older teachers (β = 0.0620), stronger for medium age teachers (β = 0.0674), and strongest for younger teachers (β = 0.0729).

## 5. Discussion

In the period of the COVID-19 pandemic, due to the large-scale lockdown of schools, students had to study online at home; meanwhile, teachers were confronted with sudden online teaching and the need to adapt to online teaching. For example, the implementation of technology in online teaching adds a certain degree of stress for teachers, and the mastery of technical pedagogical competence affects the implementation of teachers’ online teaching [5]. Teacher competence has been exposed in the process of large-scale online teaching. Meanwhile, students also have to study online, but the practical effect of online learning is a source of disagreement [98]. Therefore, the present study focused on whether teachers’ competence can support students’ online learning well, and explored how it affected teacher resilience and students’ online learning outcomes.

This study is the first to examine whether the influence of teacher competence on perceived online learning outcomes may be mediated by teacher resilience. Our findings suggested that teacher competence in online teaching and teacher resilience positively correlated with perceived online learning outcomes, and links between teacher competence and perceived online learning outcomes were mediated by teacher resilience. More details are described as follows.

### 5.1. TCOT Is Positively Related to POLO

The results of the correlational analysis and mediating analysis indicated that TCOT was positively related to POLO (H1 supported). This finding is in line with some previous studies (e.g., [23,46]). Those studies indicated that teacher competence affects student learning outcomes via influencing the behaviors of teachers in class and teacher–student interactions (i.e., teaching quality) in online teaching. Teacher competence is composed of self-efficacy, pedagogical content knowledge (PCK), beliefs, teaching enthusiasm, and so on. It has been confirmed that students whose teachers have a high level of PCK exhibit better academic performance and a higher level of motivation [99,100]. This online teaching practice in the COVID-19 pandemic places new demands on teachers’ PCK, as they need to update their PCK to effectively integrate technology into their online teaching [32]. Some studies have indicated that the effective use of technology in online teaching can help students learn efficiently [101]. Teachers with a high belief of self-efficacy can generate greater teaching engagement and higher levels of intrinsic motivation, which is also conducive to the creation of new teaching models [102]. Furthermore, teacher self-efficacy influences learners’ learning outcomes through factors that affect the quality of teachers’ teaching, such as teacher commitment and motivation. In addition, for students, teachers with a high level of enthusiasm for teaching can easily create a warm classroom atmosphere and be more motivated to provide support to students, thus, stimulating students’ learning interest and improving their learning outcomes [39,91,103]. Therefore, teachers should update their teacher competence in online teaching from different dimensions, so as to improve their online teaching quality and enable students to produce more excellent learning outcomes.

### 5.2. TCOT Is Positively Related to TR

Moreover, the positive correlation between TCOT and POLO was found in this study (H2 supported). This finding is in line with Beltman’s [60] literature review, which concluded that the development of teacher resilience is affected by personal protective factors, such as self-efficacy, enthusiasm, teaching skills, and so on. Specifically, teachers’ self-efficacy and beliefs provide support for them to overcome challenges in the teaching process [15]. Teachers with sufficient TPCK can reduce their work pressure and improve their resilience [104]. Therefore, teachers should improve and develop their teacher competence to increase their resilience in responding effectively to unexpected situations in the teaching process and to improve teacher well-being.

### 5.3. TR Is Positively Related to POLO

This study also found a negative correlation between TR and POLO (H3 supported). This result is in line with the research confirmed by Gu and Day [15]. During online teaching, teachers may experience certain stress and undesirable emotions, such as technology stress [6,53,54]). Teachers should be effective at stress relief and emotional control to avoid the transmission of bad emotions to students, thus affecting the quality of their online learning. Chou and Chou [5] showed that teachers’ stress tolerance will affect their continuance intention of online teaching. That is, if teachers do not have good resilience, sudden online teaching activities may not be carried out properly, thus affecting students’ learning outcomes.

### 5.4. TR Mediates the Relation between TCOT and POLO

In this study, the role of TR in mediating between TCOT and POLO was confirmed, and TCOT was also associated with POLO when TR was included as the mediating role (H4 supported). The effect of teacher competence in online teaching on students’ online learning outcomes is influenced by teacher resilience. That is, teachers with high competence have the resilience to face adversity and are able to cope well with the sudden transition of online teaching, thereby increasing student online engagement and learning outcomes.

Based on the findings above, we can conclude that teacher competence in online teaching and teacher resilience are two important factors that influence students’ online learning outcomes. Therefore, attention should be paid to the development of teachers’ resilience. Teachers with high levels of well-being and resilience can establish a positive atmosphere, which is the foundation for students to achieve learning outcomes. The interventions that can improve teachers’ resilience is the focus of current studies. This study focused on cultivating resilient teachers with high levels of well-being through the intervention of teacher competence in online teaching.

The COVID-19 pandemic has led to school lockdowns, and nearly half of the world’s students have had to learn online at home at some time during the pandemic [105]. This massive online teaching and learning scenario re-emphasizes the importance of technology integration into education [32,106], and how to develop teacher competence in online teaching is the focus of current studies. Teachers with high teacher competence demonstrate a high degree of resilience in responding to instructional emergencies, helping students to better adapt to online learning and to achieve the expected learning outcomes. Although teachers may have the awareness and need to develop their competence in online teaching, how to develop and enhance their competencies in many ways is what they need to focus on now.

Teacher competence can be influenced by both contextual and personal factors, with the former emphasizing the role of infrastructure, access to technology, and school support in supporting teacher competency [85]. Therefore, in addition to guiding teachers to properly perceive the important role and value of teacher competence in online teaching, schools should also provide some external support for teachers. Currently, many schools are in the process of ICT transformation in the education system. The integration of technology into education must first be systematically received by teachers and implemented in their daily teaching processes. The COVID-19 pandemic situation shows what the consequences are if teachers cannot use ICT competently. Therefore, it is very important to provide learning and training opportunities for teacher competence development to teachers.

### 5.5. TA Moderates the Direct and Indirect Relations between TCOT and POLO

An intriguing finding of this study is that the indirect relationship between TCOT and POLO through teacher resilience is moderated by TA (H5 was partly supported). In particular, we found that the indirect effect mechanism was more pronounced among younger teachers. This finding is consistent with the socio-emotional selectivity theory [107]. Young teachers have more advantages in ICT skills, which play a key role in teacher competence in online teaching, but their resilience is relatively weak. Therefore, young teachers can give full play to their advantages of competence in online teaching via improving their resilience and helping students achieve ideal online learning outcomes. On the contrary, the resilience of older teachers is stronger, but the lack of ICT skills makes it difficult to highlight their strength in resilience in the context of online learning.

In addition, TA also moderated the direct correlation between TCOT and POLO, and the positive effect of TCOT on POLO was stronger in the older teacher group. That is, compared to the higher levels of TCOT among younger teachers, the higher levels of TCOT among older teachers had a more significant effect on POLO. The possible explanation is that older teachers aged 45 and above have a high level of teaching pedagogical knowledge, although their digital competence is not so good [108]. Therefore, for older teachers, the improvement of competence in online teaching can significantly improve their teaching effectiveness. For young teachers, their teaching competence in many aspects needs to be developed. Furthermore, older teachers tend to have accumulated rich teaching experience, and experienced teachers are better able to use their teaching competence to help students achieve learning outcomes [109].

However, the moderating role of teachers’ age in the association between TCOT and TR (first stage moderation) was not found. A possible and reasonable explanation for this finding is that, to some extent, both young and old teachers who are incompetent in online teaching may experience a low level of resilience. This finding has been confirmed by previous studies. Xu et al. [17] confirmed the significant correlation between teachers’ competence and resilience among teachers of different age groups. In Kang and Yoo’s [110] study, competence was determined as a strong predictor of teachers who had 10 to 19 and 20 to 29 years of experience.

Another explanation may be that age is not the most important influencing factor for teacher competence and resilience, especially in the context of online learning. Furthermore, compared with other risks or protective factors, teachers’ age has a limited moderating effect between competence and resilience in online teaching. For instance, Kerzic et al. [111] revealed that age is not a factor that affects the use of ICT in teaching, although there are age differences in the use of ICT by teachers.

## 6. Conclusions

During the lockdown due to the COVID-19 pandemic, the implementation of online teaching has been a great challenge for teacher competence and resilience, and teachers need to develop their resilience through updating their teaching competence to support students’ online learning. The conclusions of this study provide some suggestions for the future development of teacher competence in online teaching and teacher resilience.

The results of this study showed that TCOT was positively related to TR (H2) and positively related to POLO (H1). TR was positively related to POLO (H3), and acted as a mediator between TCOT and POLO (H4). TA partly moderated the relation between TCOT, TR, and POLO (H5). Accordingly, it is not only beneficial but crucial to promote teacher resilience, while enhancing teacher competence. Meanwhile, the difference in TCOT and TR between younger and older teachers should be noted. This study suggests appropriate recommendations for teachers and schools, including how to improve the level of teacher competence in regular teaching and how to enhance teacher resilience thresholds in response to unexpected situations during teaching.

### 6.1. Implications

The contribution of this research to society is shown in the following aspects. Regarding theoretical contributions, past research has focused on the study of the correlation between teacher competency and student academic achievement or the impact of teacher resilience on student learning, but has failed to explore the associations among TCOT, TR, and POLO in online teaching and learning. In addition, research on teacher resilience has been limited to the conventional context of face-to-face teaching. The literature on teacher resilience has, therefore, been enriched by this research. Another theoretical implication of this study is that it moves away from the utilization of technology in online teaching and focuses more on the importance of people. When separated from students in time and space and facing the sudden transition to online teaching, teachers may show a certain amount of stress and anxiety. Thus, teacher resilience becomes more important in online teaching. This is why the mediating role of teacher resilience was found in this study. The third theoretical implication of this paper is that it explored the correlation between TCOT and TR from the perspective of teacher age, which makes up for the deficiency of past studies that only take age as a control variable.

A practical implication of this study is that it provides a theoretical basis and suggestions for how to train teacher competence in online teaching and integrate it into teacher education in the post-pandemic era. First of all, teachers should be competent in online teaching, especially be able to use digital technology flexibly in their online teaching. It has been pointed out that teachers’ self-efficacy is positively correlated with their digital competence [112]. Furthermore, in terms of teacher competence in online teaching and digital use, support should be provided at the organizational level in addition to advancing on an individual level. Previous research has shown that the support teachers receive from management will influence the extent to which they use technology in online teaching [113,114]. Therefore, it is necessary to improve online teaching service guarantees to support the development of teachers’ teaching competence. Specifically, schools should strengthen all kinds of online teaching platforms, network conditions, and other support for online teaching. In addition, both pre-service teachers and post-service teachers should strengthen the training of teacher competence in online teaching and build an online teaching and training system for teachers. Finally, the cultivation of teacher competence in online teaching should not only be carried out before and during teaching, but should also pay attention to the evaluation of teachers’ competence, so as to promote the precise improvement of teacher competence in online teaching with the evaluation results.

Another practical implication of this study is that it provides interventions and strategies for teachers to support their resilience and well-being. Firstly, the findings of this study indicated that teacher resilience is affected by teacher competence, so strengthening the cultivation of teacher competence in online teaching is an important factor in improving teacher resilience. On the one hand, teachers’ pedagogical knowledge and self-efficacy should be improved [115]. Enhancing teachers’ perceptions of their own pedagogical knowledge and skills can improve their resilience and well-being when facing adversities, such as the COVID-19 pandemic. On the other hand, teachers’ enthusiasm and positive beliefs are also key to their resilience and well-being. Studies have confirmed that students prefer enthusiastic teachers [65]. In addition, the existing research has also pointed out some relevant interventions to cultivate teachers’ resilience and well-being. For example, when teachers face dilemmas, cooperative problem-solving and help-seeking are common strategies to improve their resilience [116,117,118]. Meanwhile, IBSR interventions in schools are also one of the most effective ways to help teachers improve their resilience and well-being in the face of sudden dilemmas, such as the COVID-19 pandemic [119].

The final practical implication is that this study offers a reference for providing different types and degrees of online teaching instruction to teachers of different ages. For young teachers, more attention should be paid to the improvement of their resilience, so as to better leverage their advantage of their competence in online teaching. As for older teachers, competence in online teaching should be cultivated to give play to their strength in resilience.

### 6.2. Limitations and Future Study

It has been noted that both teacher competence and teacher resilience are influenced by individual factors, such as gender, age, teaching experience, and so on [76,120,121,122,123]. These were used as control variables in this study; future studies may compare different groups of teachers by gender and years of teaching during online teaching to explore the competence and resilience of different groups of teachers.

In addition, from the perspective of teachers, this study understood the online learning outcomes perceived by teachers and only took teachers as participants. In future research, the online learning outcomes should be investigated from the perspective of student self-assessment.

Finally, teacher competence is an important predictor of teaching quality and student academic performance, but this study lacks analysis of the characteristics of teacher competence in online teaching and the factors that influence it. Thus, future studies may include teacher competence in online teaching as a predictor of students’ online learning outcomes to check if its multiple linear regression can confirm the essential nature and factors of teacher competence in online teaching.

## Figures and Tables

**Table 1:** The correlational analysis results.

Variables	X	SD	POLO	TR	TCOT
TA	2.770	0.880	−0.059 **	−0.004	−0.094 **
TCOT	3.846	0.642	0.370 **	0.397 **	1
TR	3.728	0.692	0.397 **	1	
POLO	2.978	0.808	1		

Note. ** *p* < 0.01. (Source: drawn up by the authors).

**Table 2:** The mediation model of teacher resilience between teacher competence in online teaching and perceived online learning outcomes.

Predictors	TR			POLO		
	β	SE	95%CI	β	SE	95%CI
Constant	2.02 **	0.014	(1.996, 2.049)	0.79 **	0.017	(0.752, 0.818)
Gender	−0.04 **	0.004	(−0.047, −0.033)	0.01 **	0.004	(0.007, 0.023)
Years of teaching	0.013 **	0.001	(0.011, 0.016)	−0.03 **	0.002	(−0.031, −0.024)
Prior experience	0.039 **	0.002	(0.035, 0.043)	0.07 **	0.002	(0.066, 0.074)
TCOT	0.42 **	0.003	(0.413, 0.423)	0.373 **	0.003	(0.367, 0.380)
TR				0.161 **	0.003	(0.155, 0.166)
R^2^	0.162			0.161		
F	7684.945 **			6089.145 **		

Note. CI = confidence interval; β = standardized coefficient; ** *p* < 0.01. (Source: drawn up by the authors).

**Table 3:** Direct effect, indirect effect, and total effect.

	β	SE	95%CI	
			LL	UL
Direct effect	0.374	0.003	0.000	0.367
Indirect effect	0.067	0.001	0.065	0.070
Total effect	0.441	0.003	0.000	0.435

Note. CI: confidence interval; LL: lower limit; UL: upper limit. (Source: drawn up by the authors).

**Table 4:** The coefficients of the moderated-mediation analysis.

	β	SE	95%CI	
			LL	UL
**TR**				
**TA**	0.0134 **	0.0028	0.008	0.0189
**TCOT** × **TA**	−0.0031	0.0028	−0.0086	0.0025
*R*^2^ = 0.162 ** F = 5128.1647				
**POLO**				
**TA**	0.0008	0.0033	−0.0056	0.0072
**TCOT** × **TA**	0.0375 **	0.0036	0.0304	0.0445
**TR** × **TA**	−0.0136 **	0.0033	−0.0202	−0.0071
*R*^2^ = 0.1611 **				
*F* = 3821.9235				

Note. CI = confidence interval; β = standardized coefficient; ** *p* < 0.01. (Source: drawn up by the authors).

**Table 5:** Conditional indirect effect of TCOT on POLO through TR for teachers of different ages.

Different Ages of Teachers	Indirect Effect	Boot SE	LL	UL
Younger teachers (M + 1SD)	0.0729	0.0019	0.0693	0.0766
Medium (M)	0.0674	0.0013	0.0648	0.0700
Older teachers (M − 1SD)	0.0620	0.0018	0.0585	0.0657

(Source: drawn up by the authors).

## Data Availability

Some or all of the data and models that support the findings of this study are available from the corresponding author upon reasonable request.
